# Women Remain at Risk of Iodine Deficiency during Pregnancy: The Importance of Iodine Supplementation before Conception and Throughout Gestation

**DOI:** 10.3390/nu11010172

**Published:** 2019-01-15

**Authors:** Kristen L. Hynes, Judy A. Seal, Petr Otahal, Wendy H. Oddy, John R. Burgess

**Affiliations:** 1 Menzies Institute for Medical Research, University of Tasmania, Private Bag 23, Hobart, TAS 7001, Australia; Petr.Otahal@utas.edu.au (P.O.); Wendy.Oddy@utas.edu.au (W.H.O.); 2 Public Health Services, Department of Health and Human Services, Tasmanian Government, GPO Box 125, Hobart, TAS 7001, Australia; Judy.Seal@health.tas.gov.au; 3 School of Medicine, University of Tasmania, Private Bag 34, Hobart, TAS 7001, Australia; j.burgess@utas.edu.au

**Keywords:** iodine nutrition, iodine deficiency, pregnancy, supplementation, public health, pre-conception

## Abstract

In Australia, pregnant women are advised to take an iodine supplement (I-supp) (150 µg/day) to reduce risks to the foetus associated with iodine deficiency (ID). To examine the impact of this recommendation on iodine status, and to identify factors that contribute to adequacy during gestation, supplement use and Urinary Iodine Concentration (UIC) was measured in 255 pregnant women (gestation range 6 to 41 weeks) in Tasmania. The median UIC (MUIC) of 133 µg/L (Inter-quartile range 82–233) was indicative of ID, being below the 150–249 µg/L range for adequacy during pregnancy. Women taking an iodine-containing-supplement (I-supp) had a significantly higher MUIC (155 µg/L) (*n* = 171) compared to the combined MUIC (112.5 µg/L) (*n* = 84) of those who had never (120 µg/L) (*n* = 61) or were no longer taking an I-supp (90 µg/L) (*n* = 23) (*p* = 0.017). Among women reporting I-supp use, the MUIC of those commencing the recommended 150 µg/day prior to conception was significantly higher than those starting supplementation following pregnancy confirmation: 196 (98–315) µg/L (*n* = 45) versus 137.5 (82.5–233.5) µg/L (*n* = 124), *p* = 0.032. Despite recommendations for iodine supplementation pregnant Tasmanian women remain at risk of ID. Commencing an I-supp of 150 µg/day prior to conception and continuing throughout pregnancy is required to ensure adequacy. Timely advice regarding the importance of adequate iodine nutrition, including supplementation is needed to reduce the risk of irreversible in utero neurocognitive damage to the foetus.

## 1. Introduction

The risk of endemic iodine deficiency (ID) has been well characterised in the south-eastern Australian states of New South Wales (NSW), Victoria (VIC) and Tasmania (TAS) [1,2]. To address public health concerns of inadequate iodine status in Australia and New Zealand mandatory replacement of salt with iodised salt in bread was introduced in 2009 [3]. A series of urinary iodine surveys of Tasmanian school-age children, who are used as a proxy for the general population, indicates that bread fortification has successfully increased iodine status from deficiency to adequacy [4]. However, fortification has had little impact on the status of pregnant women, increasing the pre-fortification median urinary iodine concentrations (MUIC) from 76 µg/L to 86 µg/L [5], well below the World Health Organization (WHO) 150–249 µg/L range for adequacy in pregnancy [6]. Similar results have been reported elsewhere [7,8].

Adequate iodine nutrition is essential during pregnancy for foeto-maternal production of thyroid hormones required for optimal foetal neurodevelopment. The consequences of severe iodine deficiency (ID) include spontaneous abortion, stillbirth, congenital abnormalities and endemic cretinism [6]. Increasing evidence suggests that even transient, mild gestational ID has subtle negative impacts, including reductions in IQ [9], educational outcomes [10,11] and language skills [12,13].

Dietary iodine requirements rise by approximately 50% during pregnancy, driven by increased thyroid synthetic demands, increased renal iodine clearance, and transfer of iodine and thyroxine to the foetus [14]. National Health and Medical Research Council (NHMRC) guidelines recommended that the daily intake (RDI) of iodine for adolescents and adults increase from 150 µg/day to 220 µg/day during pregnancy [15]. In regions classified as mildly or borderline deficient, pregnant women may have difficulty increasing their level of iodine intake to provide sufficient nutrition for the developing foetus, and pregnant women, and in some cases women of child-bearing age, are iodine deficient despite the general population being considered sufficient [16,17].

Recognition that the current mandatory bread fortification program alone is insufficient to meet the increased demand during pregnancy, the NHMRC introduced recommendations in 2010 for pregnant and breastfeeding women, and women planning pregnancy, to take a daily iodine supplement of 150 µg/day to help meet the 220 µg/day RDI [18]. It advises supplementation “from the point of planning pregnancy” or “as soon as possible after” confirmation of pregnancy, with supplementation continuing throughout pregnancy and breastfeeding.

We report the iodine status of a group of pregnant Tasmanian women following the introduction of mandatory bread fortification and recommendations for iodine supplementation, and discuss factors contributing to adequate iodine nutrition during gestation.

## 2. Materials and Methods

WHO sentinel surveillance techniques, employed globally to assess and monitor iodine nutrition within populations [6], were used. A convenience sample of pregnant women residing in the southern Tasmanian region were recruited at Royal Hobart Hospital antenatal clinics, and via social media and conventional advertising, as part of a larger investigation (Tasmanian Women’s Iodine Nutrition Knowledge (TWINK) Study). Women at all stages of gestation were eligible. To ascertain the iodine status of women of reproductive age, a representative sample of women residing in the same local government areas as the pregnancy group were selected from the Tasmanian electoral roll. Non-pregnant, non-lactating women were eligible. Participants provided a spot urine sample for urinary iodine concentration (UIC) assessment and completed a questionnaire on enrolment. Use and dose of dietary supplements containing iodine (I-supp) during pregnancy and prior to conception was recorded. As retrospective recording of the precise time that supplementation was started is subject to recall bias, iodine supplementation start time is reported dichotomously as “Before” and “After” conception. In Australia, a large variety of over-the-counter nutritional supplements containing iodine are available in tablet, capsule and liquid form, including supplements marketed specifically for pre-conception, pregnancy and lactation, as well as many multi-vitamin formulations that include iodine. Differences in the active amount of iodine and dosage recommendations result in a range of recommended daily doses. To determine compliance with the NHMRC recommendation of 150 µg/day [18], participants were asked to provide information on the brand and the daily dose of all nutritional supplements they were taking. At the end of the enrolment visit each participant was provided with a copy of the 2010 NHMRC recommendations for iodine supplementation during pregnancy. Additional spot urine samples and information on I-supp use was collected during each remaining trimester. To determine the median UIC in this sample of women, only data collected at enrolment is reported here as it is probable that provision of the NHMRC recommendations, and indeed participation in the study, led to changes in behaviour with regard to supplement use and intake of iodine from dietary sources. Inclusion of samples collected after enrolment could give a false indication of the median UIC in this sample.

UIC was determined at the Institute of Clinical Pathology and Medical Research (Westmead Hospital, Sydney, Austrilia), which complies with IOS/IEC standard 17025, using the modified Sandell-Kolthoff reaction. Iodine status was defined using WHO classifications [6], where a MUIC between 150–249 µg/L during pregnancy is adequate; and general population adequacy is classified as a MUIC between 100–199 µg/L, with less than 20% below 50 µg/L.

Stata/SE 15.1 (StataCorp LLC, College Station, TX, USA) was used for statistical analysis. UIC is skewed, thus median and interquartile range (IQR) are presented. Means and standard deviations (SD) are used for continuous measures and percentages for categorical measures. *p*-Values were calculated using two-sample Mann-Whitney test of difference between MUIC for two groups, and Krushal-Wallis equality of populations rank test for three or more groups.

The study was approved by the Tasmanian Health and Medical Human Research Ethics Committee (#H0013336) and participants gave informed written consent.

## 3. Results

### 3.1. Iodine Status of Pregnant Women

Two-hundred and fifty-five pregnant women were recruited between March 2014 and November 2015. The MUIC at enrolment was 133 (IQR 82-233) µg/L (Table 1). MUIC was highest in the group of women who enrolled during the first trimester and lowest in the group of women who enrolled in the third trimester with the third trimester MUIC being significantly lower than the first (161 vs. 119 µg/L, *p* = 0.046).

### 3.2. Iodine Supplement Use

Two-thirds (*n* = 171) of participants, were taking an I-supp at enrolment, with 149 (87%) of these taking the recommended dose of 150 µg/day or more (Table 2). The mean daily dose of iodine from supplements was 178 (SD 67.4) µg/day and ranged between 43 to 500 µg/day. The percentage of participants using an I-Supp at enrolment was similar across all trimesters, with only a small decline in use from the first to the third trimester.

Those women still taking an I-supp at enrolment had a higher iodine status (155 µg/L, *n* = 171) than those who had never (120 µg/L, *n* = 61), or were no longer taking an I-supp (90 µg/L *n* = 23); the combined MUIC of these groups was indicative of ID (112.5 (77–190.5) µg/L, *n* = 84) and was significantly lower than the group still taking an I-supp (*p* = 0.017).

Among those still taking an I-supp, only those who started before conception, and took the recommended dose of 150 µg/day, or greater, attained adequacy. The MUIC of this group (196 (98–315) µg/L, *n* = 45) was significantly higher than that of the other three dose and timing groups combined (137.5 (82.5–233.5) µg/L, *n* = 124, *p* = 0.032).

Comparing those still taking I-supps, the decline observed in MUIC across trimesters was only evident among those who started an I-supp post-conception. In this group, the third trimester MUIC was significantly lower than the first trimester (184 vs. 98 µg/L, *p* = 0.032). Among those who took an I-supp prior to conception, the MUIC did not decline, but remained close to or within the adequate range throughout pregnancy.

### 3.3. Iodine Status of Non-Pregnant/Non-Lactating Women of Reproductive Age

Sixty-three non-pregnant, non-lactating women, aged 21–50 years, participated. Their MUIC was 79 (48–135) µg/L; 27.0% (17/63) were <50 µg/L, with 31.8% (20/63) between 100–199 µg/L, and; 81.0% (51/63) were <150 µg/L. The MUIC of women taking an I-supp (*n* = 10) fell within the adequate range, 104.5 (84–152) µg/L, but was not significantly higher than those who did not take an I-supp (73 (45–124) µg/L *n* = 53, *p* = 0.214).

## 4. Discussion

Our results indicate Tasmanian women continue to be at risk of ID in pregnancy. However, we show that iodine supplementation consistent with NHMRC recommendations [18] can improve iodine nutrition, with our findings underscoring the importance of pre-conception supplementation for ensuring adequacy throughout pregnancy.

ID has also been reported in pregnant women (MUIC 116 µg/L) across Australia in the 2011–2012 National Health Measures Survey (NHMS) [19]. By contrast, studies in some states have reported MUICs within, or close to, the adequate range (NSW 145.5 µg/L in 2011 and 166 µg/L in 2012 [20]; SA between 172 and 189 µg/L in 2011–2012 [21]). To understand the variation in MUICs in different areas of Australia, consideration must be given to factors influencing iodine status, including differences in consumption of iodine-containing foods, I-supp use, gestational stage, underlying population iodine status (particularly in women of reproductive age) and pre-conception thyroid stores.

In Tasmania, we have previously shown that bread fortification has little impact on pregnancy iodine status [5]. This study indicates that iodine supplementation can improve iodine nutrition to adequate levels. While the overall status of our sample was indicative of ID, women taking supplements in accordance with recommendations were within the adequate range. By contrast, women not taking supplements, whether pregnant or not, had MUICs comparable to those reported in pregnant women surveyed prior to the supplementation recommendations (86 µg/L) [5]. Other Australian studies report similar results. In two NSW [20] cohorts, MUICs were higher among women taking I-supps compared to those who didn’t (178 and 202 µg/L vs. 109 and 124 µg/L, respectively). In SA [21], higher MUICs, at two gestational time-points, were reported in women taking supplements (152 and 221 µg/L) compared to those who didn’t (141 and 159 µg/L).

Taking I-supps, however, does not guarantee sufficiency during pregnancy. A NSW study conducted prior to fortification and supplementation recommendations, reported higher, but less than adequate, MUIC among women taking I-supps compared to those not (115 and 72 µg/L, respectively) [22]. Similarly, studies in VIC [7] and SA [8], spanning the pre/post fortification/supplementation periods, found higher but still inadequate MUICs among those taking I-supps compared to those who didn’t (VIC: 121.5 and 64.5 µg/L; SA: 89 and 75 µg/L).

Not all groups taking I-supps reached sufficiency in our study; current I-supp consumption and dose were important predictors of adequacy. Only women still taking an I-supp at enrolment had a MUIC within the adequate range. Among those that had ceased supplementation there was no evidence of any enduring I-supp consumption effect on current iodine status, highlighting the importance of recent intake for maintenance of adequate nutrition and supporting the NHMRC recommendation for daily supplementation throughout pregnancy. We found evidence of a dose-response, with increased levels of supplementation associated with higher MUICs. In accord with the NHMRC recommendation, only those taking the suggested daily amount of 150 µg/day, or above, reached adequacy. By contrast, groups that had never or were no longer taking an I-supp had MUICs indicative of ID. Condo et al. [21] also report a positive dose-response; MUICs at two gestational time-points were higher in women taking ≥150 µg/day compared to those taking <150 µg/day (<20 weeks: 221 vs. 163 µg/L, and; 28 weeks: 187 vs. 152 µg/L). Unlike our study, both dose groups had MUICs within the adequate range.

The dose of I-supps required to assist women reach sufficiency for pregnancy will be influenced by the underlying iodine nutrition of the population. The lower the dietary intake of iodine from food sources, the greater the level of supplementation required. Surveys of school-age children indicate that mandatory fortification has led to adequate iodine nutrition levels in Tasmania [4]. While the NHMRC asserts that “through mandatory fortification, most of the Australian population will get enough iodine, meaning women of child-bearing age should enter pregnancy with adequate thyroid stores” [18], our data for Tasmanian women of reproductive age indicates this may not be the case. Applying 100 µg/L general population adequacy cut-point, the 79 µg/L MUIC with 27.0% <50 µg/L is indicative of ID, suggesting that women may be entering pregnancy with sub-optimal thyroid stores. While it can be argued that adult UICs between 60–70 µg/L are equivalent to 100 µg/L in children, due to larger urinary volume outputs in adults [23], we suggest that UICs at this level ill-prepare women entering pregnancy for the increased iodine requirements of gestation. Our finding is similar to national data, where women of child-bearing age in the 2011–2012 NHMS [19,24] had lower iodine levels than children and other adults. Although the MUICs (Australia 121 µg/L; Tasmania 105 µg/L) were within the adequate range for non-pregnant/non-breastfeeding women, the report [24] raised concerns that mandatory fortification may not be meeting the needs of women of child-bearing age. Concerningly, 18.3% of women across Australia had UICs <50 µg/L, which approaches the 20% cut-point indicative of insufficient iodine intake.

Further evidence that Tasmanian women are entering pregnancy with inadequate thyroid stores is provided by the changes in MUIC as pregnancy progresses. Studies show that UICs, which increase early in pregnancy, follow two patterns as pregnancy progresses, depending upon the underlying population iodine status [25]. In populations where ID exists MUICs in the 2nd and 3rd trimesters decline, often falling below adequate levels. The decline is less marked in iodine sufficient populations with MUICs usually remaining within the optimal range throughout gestation. The pattern observed in our study is indicative of a population with inadequate iodine nutrition. However, among the small number of women who began supplementation prior to conception no decline was evident, with MUICs remaining adequate across trimesters. These observations support the notion that adequate pre-conception thyroid stores, whether attained via iodine from foods, I-supps, or a combination, are essential for maintaining iodine levels throughout pregnancy.

Women in our study who started supplementation after pregnancy did not maintain adequate levels as gestation progressed, suggesting that although supplementation after pregnancy improves UICs, it may not be enough to replenish or maintain thyroid stores that are inadequate prior to conception. A recent UK study found that inadequate pre-conception iodine status (MUIC 108.4 µg/L, 17.8% <50 µg/L) was positively associated with poorer cognitive function in the offspring [26]. Given many pregnancies are unplanned, and women may be unaware until well into the first trimester, it is vital that women of child-bearing age have adequate pre-conception iodine nutrition to ensure adequacy for foetal neurodevelopment in early gestation. In populations where iodine intake from dietary sources is inadequate, pre-conception supplementation may improve thyroid stores prior to pregnancy.

Adequate dietary iodine preconception is also important for maintenance of pregnancy, with miscarriage rates known to be higher in regions of severe iodine deficiency [27]. While we found no significant difference in the MUIC of women who had previously had a miscarriage compared to those who had not, the percentage of women (31.4%) reporting ever having had a miscarriage was higher than expected (up to 25%) in Australia [28]. While it is beyond the scope of this study to explain the high level of miscarriage in our sample, the long-standing existence of iodine deficiency in the Tasmanian population may be an important factor. However, it is not unreasonable to expect some recruitment bias in our sample, with women who have previously had an unexpected outcome in pregnancy being more likely to participate in a study of pregnancy knowledge, because of a heightened interest fuelled by their past experience.

Our study has some limitations. To obtain precise group MUICs from spot urine samples, approximately 125 samples are required to estimate iodine levels with 95% confidence and ±10% precision [29]. Although our results are consistent with other studies, care must be taken when interpreting MUICs of small sub-groups. Furthermore, although it is appropriate to use spot samples to determine the median value of a group, UIC is not ideal for classifying individuals, as a single sample may not be indicative of usual status. UIC can vary day-to-day depending upon dietary intake, and while a spot sample reflects ingested iodine over the past 24-h, it can be influenced by circadian rhythm and post-meal peaks [30]. For use as an individual measure a minimum of 10 samples are recommended [29,31]. Although women in our study provided samples each trimester it was not feasible to collect more and given the confounding effects of gestational stage on UIC taking the average over trimesters is not advisable. Lack of a gold-standard measure of individual status means caution must be exercised when results derived from techniques designed for assessing populations are used to develop public health advice for individuals. Caution is also advised regarding the generalisability of our findings to all pregnant women in Tasmania, or elsewhere in Australia, as recruitment of a volunteer sample may result in selection bias and lack of representativeness. Although our research has previously shown no link between measures of socio-economic status and iodine nutrition [32], our sample was slightly younger than the average pregnant women in Australia (29.3 versus 30.3 years [33]) and more likely to have completed schooling beyond Year 10 compared to Tasmania women of childbearing age [34].

Despite these limitations, it is concerning that in our sample only a small percentage of individuals had UICs within the adequate range. While a single UIC may not indicate usual status, even transient inadequate iodine consumption during pregnancy can have detrimental impacts on the fast-developing foetus. We have demonstrated that even mild maternal ID, defined as a UIC <150 µg/L at one point during pregnancy, is associated with deficits in educational outcomes that persist into adolescence [11]. Globally, epidemiological studies have linked transient maternal hypothyroidism at crucial times of foetal neurodevelopment to impaired cognitive [9] and psychomotor development [35], behavioural difficulties [36], and abnormal brain morphology [37], with a recent meta-analysis also suggesting that maternal hypothyroxinemia during gestation is associated with lower non-verbal and verbal IQ in the offspring [38]. A randomised controlled trial is needed to facilitate a rigorous examination of the impact of I-supps given a Cochrane review of past trials found “… there was insufficient data for any meaningful conclusions on the benefits and harms of routine iodine supplementation in women before, during or after pregnancy” [39]. Despite an absence of more robust measures, our study adds to a growing body of evidence suggesting that, even in regions classified as iodine sufficient, women may remain at risk of gestational ID if their intake is inadequate prior to conception and they don’t supplement throughout pregnancy. Education about iodine nutrition and the current NHMRC recommendations for supplementation before and during pregnancy to reduce the risk of irreversible long-term, yet preventable, impacts of gestational ID on offspring is needed.

## 5. Conclusions

Despite the introduction of public health measures to address ID in Australia, including mandatory fortification of bread with iodised salt and a recommendation for daily iodine supplementation prior to and throughout pregnancy and breastfeeding, our study indicates that women continue to be at risk of ID during pregnancy. Adequate iodine status was only observed among women who commenced iodine supplementation at the recommended dose prior to conception and continued throughout pregnancy. Timely advice about adequate maternal iodine nutrition, including supplementation, is necessary to reduce the risk of neurocognitive damage to the foetus.

## Figures and Tables

**Table 1:** Characteristics of pregnant women (*n* = 255) at enrolment and associated group median urinary iodine concentration (MUIC).

Characteristic	Mean (Standard Deviation) and Range, or Percentage (*n*/*N*)	MUIC ^1^ (IQR) µg/L	*p*-Value ^2^
All participants		133 (82–233)	
Age (years)	29.3 (6.0) 15–45		
Gestational Age (weeks)	21.1 (8.3) 6–41		
Trimester:			
T1 (0–13 weeks)	16.5% (42/255)	**161 (92–233)**	
T2 (14–26 weeks)	56.5% (144/255)	137.5 (81.5–243)	
T3 (≥27 weeks)	27.0% (69/255)	119 (78–185)	0.115
Highest Level of Education Completed:			
Year 10 or less	19.8% (50/253)	137.5 (82–247)	
>Year 10	80.2% (203/253)	129 (82–233)	0.851
First Pregnancy:			
Yes	36.0% (91/253)	111 (79–222)	
No	64.0% (162/253)	140 (86–236)	0.206
Intention to breastfeed:			
Yes	89.6% (223/249)	121 (87–233)	
No	10.4% (26/249)	133 (81–235)	0.765
Previous miscarriage:			
Yes	31.4% (77/245)	122 (81–235)	
No	68.6% (168/245)	137.5 (82.5–231.5)	0.793
Number of Children			
(excluding current pregnancy):			
0	43.3% (106/245)	112 (79–222)	
1	33.1% (81/245)	140 (83–243)	
2	16.3% (40/245)	**152 (87.5–226)**	
3–6	7.4% (18/245)	**175.5 (95–398)**	0.455

^1^ MUICs within the adequate range (150–249 µg/L) are shown in bold; ^2^ *p*-values for Two-sample Mann-Whitney test of difference in UIC between two groups, or Krushal-Wallis equality of populations rank test for difference in UIC for three or more groups.

**Table 2:** Iodine status of pregnant Tasmanian women categorised by iodine supplement use, dose and timing, and trimester at enrolment. Defined by median urinary iodine concentration (MUIC) and by percentage and number <150 µg/L.

Category	MUIC ^1^ (IQR) µg/L	*p*-Value ^2^	% <150 µg/L ^3^ (*n*)
**All participants (*n* = 255)**	133 (82–233)		55.3% (141)
I-Supp Use:			
Never (*n* = 61)	120 (79–207)		65.6% (40)
Ever (*n* = 194)	135 (85–235)	0.208	52.1% (101)
No longer taking (*n* = 23)	90 (70–151)		73.9% (17)
Still taking (*n* = 171)	**155 (86–247)**	**0.020**	49.1% (84)
**Among those still taking an I-supp (*n* = 171)**			
Dose ^4^:			
<150 µg/day (*n* = 20)	104 (80.5–243.5)		55.0% (11)
=150 µg/day (*n* = 93)	**153 (90–233)**		49.5% (46)
>150 µg/day (*n* = 56)	**182 (86–303)**	0.350 ^5^	44.6% (25)
Timing:			
After Conception (*n* = 123)	135 (82–234)		52.0% (64)
Before Conception (*n* = 48)	**193 (97.5–314.5)**	**0.046**	41.7% (20)
Dose ^4^ and Timing:			
After Conception & <150 (*n* = 18)	104 (78–304)		55.6% (10)
After Conception & ≥150 (*n* = 104)	140 (84–233.5)		51.0% (53)
Before Conception & <150 (*n* = 2)	131 (95–167)		50.0% (1)
Before Conception & ≥150 (*n* = 45)	**196 (98–315)**	**0.032 ^6^**	40.0% (18)
By Trimester:			
I-supp started After Conception (*n* = 123):			
T1 (0–13 weeks) (*n* = 21)	**184 (100–235)**		33.3% (7)
T2 (14–26 weeks) (*n* = 71)	**158 (81–244)**		49.3% (35)
T3 (≥27 weeks) (*n* = 31)	98 (77–161)	0.068	71.0% (22)
I-supp started Before Conception (*n* = 48):			
T1 (0–13 weeks) (*n* = 9)	144 (85–209)		55.6% (5)
T2 (14–26 weeks) (*n* = 27)	**221 (114–373)**		33.3% (9)
T3 (≥27 weeks) (*n* = 12)	**162.5 (86–266)**	0.329	50.0% (6)

^1^ Median urinary iodine concentration (MUIC) and Interquartile Range (IQR) reported. MUICs within the adequate range shown in bold; ^2^ *p*-values for Two-sample Mann-Whitney test of difference in UIC between two groups, or Krushal-Wallis equality of populations rank test for difference in UIC for three groups. *p*-Values less than 0.05 are shown in bold; ^3^ Refers to the percentage (and number) of participants with a UIC less than 150 µg/L; ^4^ Dose information unavailable for *n* = 2 participants; ^5^ For Dose, the groups compared are <150 and ≥150 (i.e., =150 and >150 combined); ^6^ For Dose and Timing, the groups compared are the last category (Before Conception & ≥150) vs. the first three categories combined (MUIC 137.5 (82.5–233.5)) (i.e., After Conception & <150; After Conception & ≥150, and; Before Conception & <150).
